# Cardiovascular Biomarkers’ Inherent Timescales in Mental Workload Assessment During Simulated Air Traffic Control Tasks

**DOI:** 10.1007/s10484-020-09490-z

**Published:** 2020-10-04

**Authors:** Thea Radüntz, Thorsten Mühlhausen, Marion Freyer, Norbert Fürstenau, Beate Meffert

**Affiliations:** 1 Unit Mental Health and Cognitive Capacity, Federal Institute for Occupational Safety and Health, Nöldnerstr. 40-42, 10317 Berlin, Germany; 2 Institute of Flight Guidance, German Aerospace Center, Brunswick, Germany; 3 Department of Computer Science, Humboldt-Universität zu Berlin, Berlin, Germany

**Keywords:** Mental workload, Heart rate, Heart rate variability, Psychophysical methods

## Abstract

One central topic in ergonomics and human-factors research is the assessment of mental workload. Heart rate and heart rate variability are common for registering mental workload. However, a major problem of workload assessment is the dissociation among different workload measures. One potential reason could be the disregard of their inherent timescales and the interrelation between participants’ individual differences and timescales. The aim of our study was to determine if different cardiovascular biomarkers exhibit different timescales. We focused on air traffic controller and investigated biomarkers’ ability to distinguish between conditions with different load levels connected to prior work experience and different time slots. During an interactive real-time simulation, we varied the load situations with two independent variables: the traffic volume and the occurrence of a priority-flight request. Dependent variables for registering mental workload were the heart rate and heart rate variability from two time slots. Our results show that all cardiovascular biomarkers were sensitive to workload differences with different inherent timescales. The heart rate responded sooner than the heart rate variability features from the frequency domain and it was most indicative during the time slot immediately after the priority-flight request. The heart rate variability parameters from the frequency domain responded with latency and were most indicative during the subsequent time slot. Furthermore, by consideration of biomarkers’ inherent timescales, we were able to assess a significant effect of work experience on heart rate and mid/high frequency-band ratio of the heart rate variability. Results indicated that different cardiovascular biomarkers reveal different inherent timescales.

## Introduction

One central topic in ergonomics and human-factors research is the assessment of mental workload. Mental workload describes the cognitive demands required in order to solve a task and relates them to the cognitive resources available (Eggemeier et al. 1991; Kahneman 1973; Wickens 2002; Xie and Salvendy 2000). Following this definition, it can be expected that registration and evaluation of mental workload is particular important in order to minimize errors and enhance human performance. Simultaneously, several studies indicated that mental workload can be linked to mental health (Zoer et al. 2011; Klonowicz 1995).

These issues particularly arise in occupations with high cognitive demands and responsibility. In such cases, employees have to maintain their performance even under difficult situations. Air traffic control is an example of such safety–critical environment. Here, inappropriate workload can have a number of negative consequences not only on employee’s health (Zoer et al. 2011; Katsamanis Karavidas et al. 2010; Klonowicz 1995; Kompier and Kristensen 2001; Landsbergis et al. 2003; NIOSH 2002) but also on the safety of persons (Katsamanis Karavidas and Lehrer 2009; Parasuraman et al. 1993; Sträter 2001). Thus, a valid and reliable method for registering mental workload is urgently needed.

Researchers have been studying various methods since decades. They distinguish between subjective and objective methods for measuring workload. While the subjective methods register participant’s experienced workload by means of questionnaires, the objective methods rely upon registration and analysis of performance or bio-physiological data. The main advantages of subjective methods are the relatively low data acquisition effort and the high user acceptance. Their main drawback is that they suffer from subjective distortion. They are influenced by memory lapses as the experienced workload took place at some time in the past and they are subject to social desirability bias as the individuals think that they are expected to provide a certain kind of answer (Lehrer et al. 2010; Radüntz 2017). The questionnaire’s items may not be readily understood or participants may lack the ability to introspect. What is more, they do not allow for fine-grained temporal sampling on the time scale of seconds and can alter the current workload state, e.g., if during a monotonous task the participant becomes activated by answering questions (Radüntz 2017). The analysis of biosignals as objective measures offer the possibility to continuously determine mental workload. They do not interfere with participant’s current workload state as they can be obtained on-the-fly during task execution. Their main issue is that user acceptance may be impaired because of the complexity of the registration system. However, recent developments in mobile sensor technology promise small, lightweight, and wireless systems (Radüntz 2017). Bio-physiological data include, among others, cardiovascular biomarkers which are easy to assess and were frequently used to analyse cardiovascular activity under a wide range of experimental conditions (Karavidas et al. 2006; Lehrer et al. 2010). The heart rate and the heart rate variability (HRV) are the most prominent biomarkers.

In most cases HRV is characterized in the frequency domain by means of various spectral features. According to the definitions by Mulder et al. (2004), the frequency range can be categorized in three bands: the low-frequency (LF: 0.02–0.06 Hz), mid-frequency (MF: 0.07–0.14 Hz), and high-frequency (HF: 0.15–0.4 Hz) bands. In 1981, Mulder and Mulder (1981) found that spectral power of the HRV between 0.02 and 0.20 Hz was in association with non-linear processes of body temperature and blood pressure control while spectral power between 0.20 and 0.40 Hz was related to the respiratory activity (parasympathetic control mechanisms). Under mental load the total spectral power decreased, whereby the spectral power between 0.02 and 0.20 Hz was particularly affected and contributed about 80% to the total spectral energy. The frequency band between 0.06 and 0.14 Hz was found to be related to the dynamic control of the mean arterial blood pressure. This band was relatively independent of respiratory rate and depth changes. In general, the HF component was associated with the parasympathetic system and the MF component with the sympathetic system although disagreement exists regarding the latter (Malik et al. 1996; Heathers 2014; Quintana and Heathers 2014).

Numerous studies showed that the spectral power around 0.1 Hz varied with the mental workload. Amplitudes around 0.1 Hz decreased under mental workload due to the controlled information processing (Mulder 1992; Jorna 1992, 1993; Veltman and Gaillard 1993; Rivecourt et al. 2008) although there were also studies with contradictory results (Cinaz et al. 2013; Nagasawa and Hagiwara 2016). Nickel and Nachreiner (2003) have thoroughly investigated the sensitivity and diagnostics of the 0.1 Hz component using various tasks and varied difficulty levels. Their findings argued against the applicability of the 0.1 Hz component for the determination of mental workload. Furthermore, significant correlations between subjective ratings and task demands with spectral components of HRV were rare while heart rate showed better correlation results (Gao et al. 2013). In order to assess the workload of pilots during work, Jorna (1993) used heart rate variability (MF: 0.07–0.14 Hz) as a real-time and continuous measurement method for a dynamic task environment. The author found that mental states and dynamic responses to variations in workload were reliably detected by heart rate variability parameter that decreased with increasing load. Rivecourt et al. (2008) investigated to which degree heart rate, HRV, and eye activity represent momentary changes in mental effort. The heart rate increased, while HRV (MF: 0.07–0.14 Hz), dwell time, and fixation duration decreased with increasing task demand.

Taken together, one of the major problems of workload assessment is the dissociation among different workload measures. One potential source of error in prior research could be the almost total disregard of their inherent timescales. In a recent article, Hancock (2017) suggested that “each of these methods (and each of their component elements) possess their own inherent time-scale” and that dissociation could be subjected to such temporal differences. He stated that participants’ workload responses might be nonlinear, complex, and time varying and concluded that associations in one selected time slot could become dissociations in another. In his roadmap for future workload research, Hancock emphasized the importance of investigation of such dissociations between measurement methods as a challenge.

In order to emphasize the topic of our paper, we briefly explain what we mean when talking about inherent timescales of biometric signals. The idea behind the concept of inherent timescales is that measures might react to task load with a different latency. These latency issues may be responsible for possible dissociations when comparing measurement values from the same time slot and can lead to contradictory results. Thus, investigation of biomarkers’ inherent timescales would be beneficial in order to prevent misleading conclusions and better understand the underlying effects. This means that different time slots should be consulted for the evaluation of mental workload and in particular, the evaluation of workload arising from critical events should consider time slots a few minutes after the critical event.

To the best of our knowledge there exists only one study that addressed this gap. Muñoz-de-Escalona and Cañas (2019) investigated temporal differences between methods’ timescales and found that workload measured by subjective methods reacted sooner than the physiological response of pupil size, in particular during high-demand peaks. The latency of the pupil-size respond was 5 min after the high-demand peak. The authors stated that “while some measures could reflect mental workload within seconds, others could show longer latency between task-load changes and mental workload index reflection” ‘. As further research objective they outlined the investigation of different physiological indicators regarding their particular timescale.

Furthermore, Jóhannsdóttir et al. (2018) argued that individual differences affecting workload measured by cardiovascular reactivity have often been neglected. Taking into account that individual differences could interact with cardiovascular biomarkers’ inherent timescales the existing gap in literature even increases. To conclude, still missing are not only studies about biomarkers’ inherent timescales but also studies about the interrelation between participants’ individual differences such as work experience and timescales.

The main aim of our study was to determine if there were differences in cardiovascular biomarkers’ ability to distinguish between different load levels connected to different time slots. We also aimed to investigate the effect of participants’ prior work experience on the obtained cardiovascular biomarkers related to their inherent timescale and load level.

To this end, we formulated the following three research questions:

1. Are cardiovascular biomarkers able to assess workload differences that arise from different traffic-volume and extraordinary-event conditions during the time slots immediately after the possible event?
2. Do different cardiovascular biomarkers exhibit different inherent timescales in mental workload assessment?
3. By consideration of biomarkers’ inherent timescales, are we able to assess a significant effect of work experience on workload?

## Materials and Methods

### Research Design

We focused on air traffic controller’s working position for arrival management and conducted a study in a simulator. The Federal Institute of Occupational Safety and Health (BAuA) in Berlin was in charge of the project. The study was conducted at the Air Traffic Management and Operations Simulator (ATMOS) of the German Aerospace Center (DLR) in Braunschweig. During the investigation, air traffic controllers interacted with pseudo pilots who simulated the cockpit crews. According to Averty et al. (2004) variations in mental workload of air traffic controllers were mainly induced by the traffic volume but might also arise by unexpected events. Thus, our simulation scenarios differed regarding two factors: traffic load and exceptional event.

The traffic-load factor consisted of four levels and varied according to the number of aircraft per hour (ac/h): 25 ac/h, 35 ac/h, 45 ac/h, and 55 ac/h. The event factor consisted of a request for a flight that should be prioritized because of a sick passenger on board. The priority-flight request could occur around the 11th minute or not. In the following, we refer to it also as priority-flight event. Both factors led to eight scenarios with a duration between 20 and 25 min. A more detailed description of our research design and implementation of the scenarios can be found in Mühlhausen et al. (2018).

### Procedure and Study Participants

21 participants in the age between 22 and 64 years participated in our study (2 female, 19 male, mean age 38 ± 11). They were coming from various airports and revealed different work positions (i.e., 13 approach controllers, 3 tower controllers, and 5 employees of the DLR), work demands, and work experience (Table 1). However, all of them were able to handle the arrival management simulation. Participants were pre-screened for co-occurring medical issues and only healthy participants were selected for the investigation.

**Table 1:** Demographic table separating the study participants in the different work positions with the addition of years of experience and age (mean (M), standard deviation (SD), minimum (min), and maximum (max) number of years)

Work positions		M	SD	Min	Max
Approach controllers, N = 10	Age	35.3	8.6	24	47
	Years of experience	10.9	8.7	0.5	23.0
Tower controllers, N = 6	Age	47.2	11.7	35	64
	Years of experience	20.0	10.3	5	33.0
Employees of the DLR, N = 5	Age	35.0	12.1	22	49
	Years of experience	4.7	5.5	0.0	14.3

Study participants completed the above-mentioned eight traffic scenarios in randomized order within two consecutive days. The first day started at noon with an introductory session where participants completed demographic questionnaires. They were briefed regarding the research goals and experimental procedure of the following 2 days. The briefing was followed by a training session at the simulator. Once they had a clear understanding of how everything worked, the main experiment started. Four of the simulation scenarios were presented on the first day, the remaining four were conducted on the second day until noon.

All of the investigations acquired were approved by the local review board of the BAuA and were carried out with the adequate understanding and written consent of the participants.

### Cardiovascular Biomarkers

For assessing cardiovascular biomarkers indicative for mental workload, we registered the pulse signal by means of a plethysmography pulse sensor at the earlobe. We used g.tec’s g.PULSEsensor and coupled it with g.tec’s mobile amplifier g.Nautilus. For signal registration we employed g.tec’s g.Recorder software. Logging of the priority-flight events was done using g.tec’s g.TRIGbox. Biosignal processing was done in Matlab.

For scenarios with a priority-flight request we considered a pulse-signal segment of 3 min starting from the request time point. For scenarios without priority-flight request we considered the pulse-signal segment from the same time slots. In the following, we refer to both of them as event slots. In order to examine possible latency effects of the cardiovascular biomarkers related to our second research question, we also considered the pulse-signal segments from minute 17 to 20 of all scenarios. These time slots are referred in the following as post slots.

In particular, we had to differentiate between the window length needed for the calculation of the HRV features and the window-starting time chosen for the evaluation of biomarker’s inherent timescales for workload assessment. The window length of 3 min was related to the recommendations by Malik et al. (1996) and aimed at assuring the stability of the signal, in particular regarding the calculation of the low-frequency band power of the HRV features. Thus, the time slot for the event slot is obviously the segment of 3 min starting from the request time point. For choosing appropriate time points for the evaluation of biomarker’s inherent timescales for workload assessment, we considered the temporal characteristics of the priority-flight request. Evaluation of event duration (i.e., request time point until handover of the priority-requesting aircraft to the tower controller) over all participants and scenarios indicated a mean value of 7 min. We concluded that possible latency effects, as connected to biosignal’s inherent timescales, should become visible after these 7 min at the latest. Consistent to the window length needed and used for the event slots, we evaluated the remaining 3 min until the end of the scenarios that resulted in post-slot segments from minute 17 to 20.

The pulse signal was windowed with a Hamming function and filtered with a bandpass filter (order 100) between 0.5 and 3.5 Hz. Next, peak detection was performed in order to gain the heart rate and the inter-beat intervals. Artifacts were automatically detected by means of statistical analysis, corrected using linear interpolation of the values at neighbouring points, and equidistantly resampled with a time resolution of 0.5 s. Heart rate was determined in beats per minute in the time domain. For evaluation of heart rate variability biomarkers in the frequency domain, we calculated the fast Fourier transform of the inter-beat interval signal from each time slot that had been previously windowed with a Hamming window. Workload-relevant frequency-band powers were computed related to Mulder (1992) for the low-frequency (LF: 0.02–0.06 Hz), mid-frequency (MF: 0.07–0.14 Hz), and high-frequency (HF: 0.15–0.4 Hz) bands. Next, we divided the LF and MF absolute band-power values by the HF-band power. Finally, for achieving a normal distribution for the further analysis, we computed the logarithms of the LF/HF and MF/HF ratios. In the following, we refer to them as lg(LF/HF) and lg(MF/HF). Eventually, we had a set of three cardiovascular biomarkers: heart rate, lg(LF/HF), and lg(MF/HF). Nevertheless, for the interested reader, we provided additional information about further analysis results for the LF, MF, and HF band powers as well as for the standard deviation of normal-to-normal (heart rate) intervals (SDNN) as a further time domain parameter in the Appendix.

### Statistical Analysis

As prerequisite for the investigation of biomarkers’ inherent timescales, we firstly investigated the ability of various cardiovascular biomarkers to assess mental workload in the time slot immediately after a possible event as stated by our first research question. We carried out three analyses of variance (ANOVA) for the event slots in order to find out which cardiovascular biomarker was able to assess air traffic controllers’ workload arising from the traffic volume, the occurrence of an exceptional event, and the interaction between both at an early stage. The dependent variable was either the heart rate, lg(LF/HF), or lg(MF/HF) values from the event slots. For each ANOVA we utilized a repeated-measures design with two within-subject factors (two levels for the priority-flight request factor and four levels for the traffic-volume factor). General differences between the levels were examined and tested with a post hoc test (Bonferroni corrected). For testing the differences between priority-flight and no priority-flight event on each traffic-volume level, we used four t-tests for each biomarker and adjusted the values accordingly.

We then looked at biomarkers’ behaviour during the post slots to answer our second research question that was concerned with biomarkers’ inherent timescales in mental workload assessment. We conducted three additional repeated-measures ANOVAs with the same factors and dependent variables as described above but now for the post slots. Calculation of separate repeated-measures ANOVAs for event and post slots was chosen as we were primarily interested to assess possible main effects of the two task-load factors during the time slots.

Finally, we addressed the issue of biomarkers’ inherent timescales in relation to participants’ prior work experience. In order to cluster the participants in two groups by work experience, we took the median of working years of our sample. This yielded 9 participants with work experience under 11 years (referred to as less experienced) and 12 participants with work experience over or equal 11 years (referred to as highly experienced). Our third research question was examined using six mixed-factorial ANOVAs. The between-subject factor for all of them was air traffic controllers’ work experience. Three ANOVAs were carried out for the event slots and three for the post slots. The dependent variables, within-subject factors, and levels were identical with those mentioned above. Similarly, we utilized a repeated-measures design and examined the differences with post hoc tests (Bonferroni).

In case of a significant between-subject factor, general group differences were analysed for each scenario by means of one-factorial ANOVAs with work experience as factor. The dependent variables were the equivalent cardiovascular biomarkers for each of the eight scenarios for the event and post slots, respectively. In order to assess work-experience differences related to the above mentioned within-subject factors, we calculated two-factorial ANOVAs with repeated-measures design for each work-experience group and examined the levels with Bonferroni-corrected post hoc test. This was done for event and post slots, respectively.

Statistical calculations were conducted using SPSS and the significance threshold was set at .05.

## Results

### Cardiovascular Biomarkers During Event Slots

Results of the ANOVAs for the cardiovascular biomarkers from the event slots, each with the two within-subject factors traffic-load and priority-flight request, are summarized in Table 2.

**Table 2:** Results of the ANOVAs for the cardiovascular biomarkers across simulation conditions for the event and post slots

		F	p	η^2^
Event slots				
Traffic load	Heart rate	6.352	**.001**	.241
	lg(LF/HF)	2.645	.057	.117
	lg(MF/HF)	2.046	.117	.093
Priority-flight request	Heart rate	6.505	**.019**	.245
	lg(LF/HF)	3.922	.062	.164
	lg(MF/HF)	0.460	.505	.022
Traffic load and priority-flight request	Heart rate	1.345	.268	.063
	lg(LF/HF)	1.594	.200	.074
	lg(MF/HF)	0.307	.820	.015
Post slots				
Traffic load	Heart rate	2.597	.061	.115
	lg(LF/HF)	6.870	**.001**	.256
	lg(MF/HF)	2.075	.113	.094
Priority-flight request	Heart rate	0.726	.404	.035
	lg(LF/HF)	5.146	**.035**	.205
	lg(MF/HF)	8.755	**.008**	.304
Traffic load and priority-flight request	Heart rate	2.764	**.050**	.121
	lg(LF/HF)	0.046	.987	.002
	lg(MF/HF)	0.702^a^	.507	.034

Statistically significant values (*p* ≤ 0.05) are given in bold

Values of .001 are actually p ≤ .001

^a^Indicates Mauchly’s test of sphericity was significant (p < .05) and a Greenhouse–Geisser correction was made to degrees of freedom

Regarding traffic load, heart rate was the only cardiovascular biomarker able to significantly differentiate between conditions. Bonferroni corrected post hoc tests showed significant differences between the low-traffic scenario with 25 ac/h and the 45 and 55 ac/h scenarios. Thereby, the heart rate increased with increased traffic.

Similarly, during the event slots, the impact of the priority-flight event became significant for the heart rate that was increased during scenarios with priority-flight request. In order to evaluate the effect for each traffic-load level separately, we computed t-tests and adjusted the values by means of Bonferroni correction. None of the t-tests could reach significance.

**Fig. 1:**
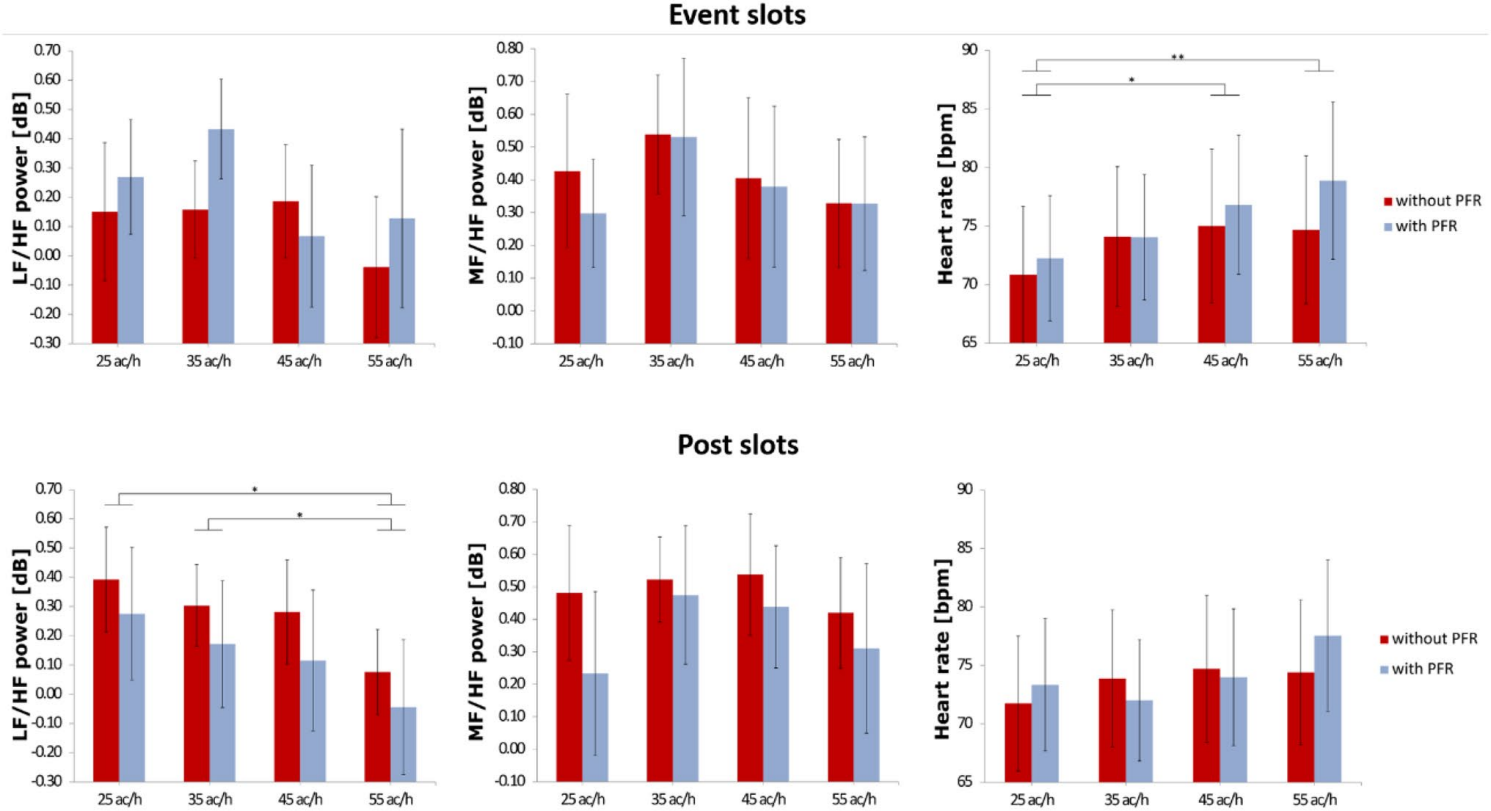
Average values of cardiovascular biomarkers over 21 participants across simulation conditions of different traffic loads (ac/h: aircraft per hour) with and without priority-flight request (PFR) for event slots (top row) and post slots (bottom row): lg(LF/HF) (left), lg(MF/HF) (center), and heart rate (right) (Bonferroni corrected post hoc tests: ***p ≤ .001; **.001 < p ≤ .01; *.01 < p ≤ .05; error bars indicate 95% confidence interval)

Finally, none of the cardiovascular biomarkers revealed an interaction effect between both factors. Figure 1 shows the results on the top row.

### Cardiovascular Biomarkers During Post Slots

**Fig. 2:**
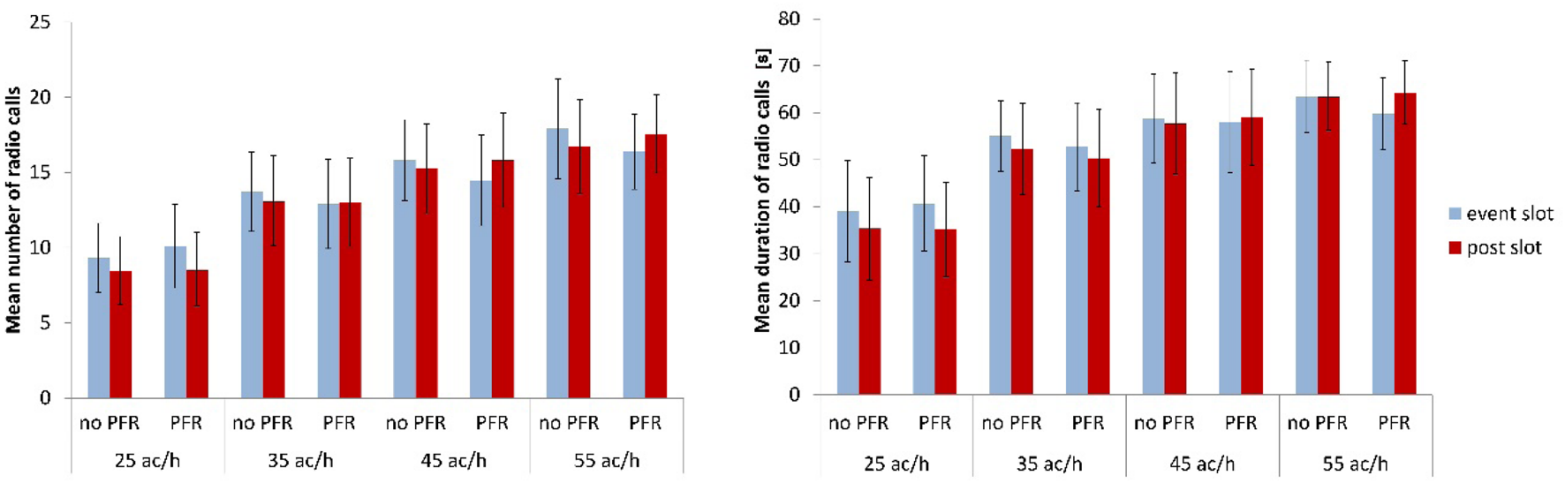
Number of radio calls (left) and radio-call duration (right) in each time slot averaged over participants for the eight scenarios of different traffic load (ac/h: aircraft per hour) with and without priority-flight request (PFR)

For investigating cardiovascular biomarkers’ inherent timescales related to our second research question, we firstly proved if slot conditions were similar. In particular, we examined if the amount of speaking between the event and post slots was comparable. This was necessary because the high-frequency band (i.e., above 0.15 Hz) is connected to respiratory activity (Mulder 1992; Jorna 1992) and influenced by speaking. We were aware that HF-band power needed for calculation of the ratios could be affected and thus, alter our results. For the sake of correctness, we calculated for each participant the number of radio calls as well as their duration for both time slots. Following, we conducted paired t-tests and adjusted the values by means of Bonferroni correction. Figure 2 shows the results. None of the t-tests became significant. This fact encouraged us to continue with our further analysis.

Results of the ANOVAs for the cardiovascular biomarkers during the post slots, with the same two within-subject factors as for the event slots above, are summarized in Table 2 and shown in Fig. 1.

During the post slots, it was only the lg(LF/HF) that was able to significantly differentiate between conditions with different traffic loads. Bonferroni corrected post hoc tests showed significant differences between the low traffic scenarios with 25 and 35 ac/h and the high-traffic scenario with 55 ac/h. Thereby, lg(LF/HF) decreased with increased traffic load.

However, during the post slots the impact of the priority-flight event became significant for the lg(LF/HF) and lg(MF/HF). Both were decreased for scenarios with a priority-flight request during the post slots. In order to evaluate the effects for each traffic-load level, we computed t-tests and adjusted the values by means of Bonferroni correction. However, none of the t-tests became significant.

Finally, the heart rate revealed a week interaction effect between both factors.

### Cardiovascular Biomarkers’ Inherent Timescales Related to Participants’ Work Experience

**Fig. 3:**
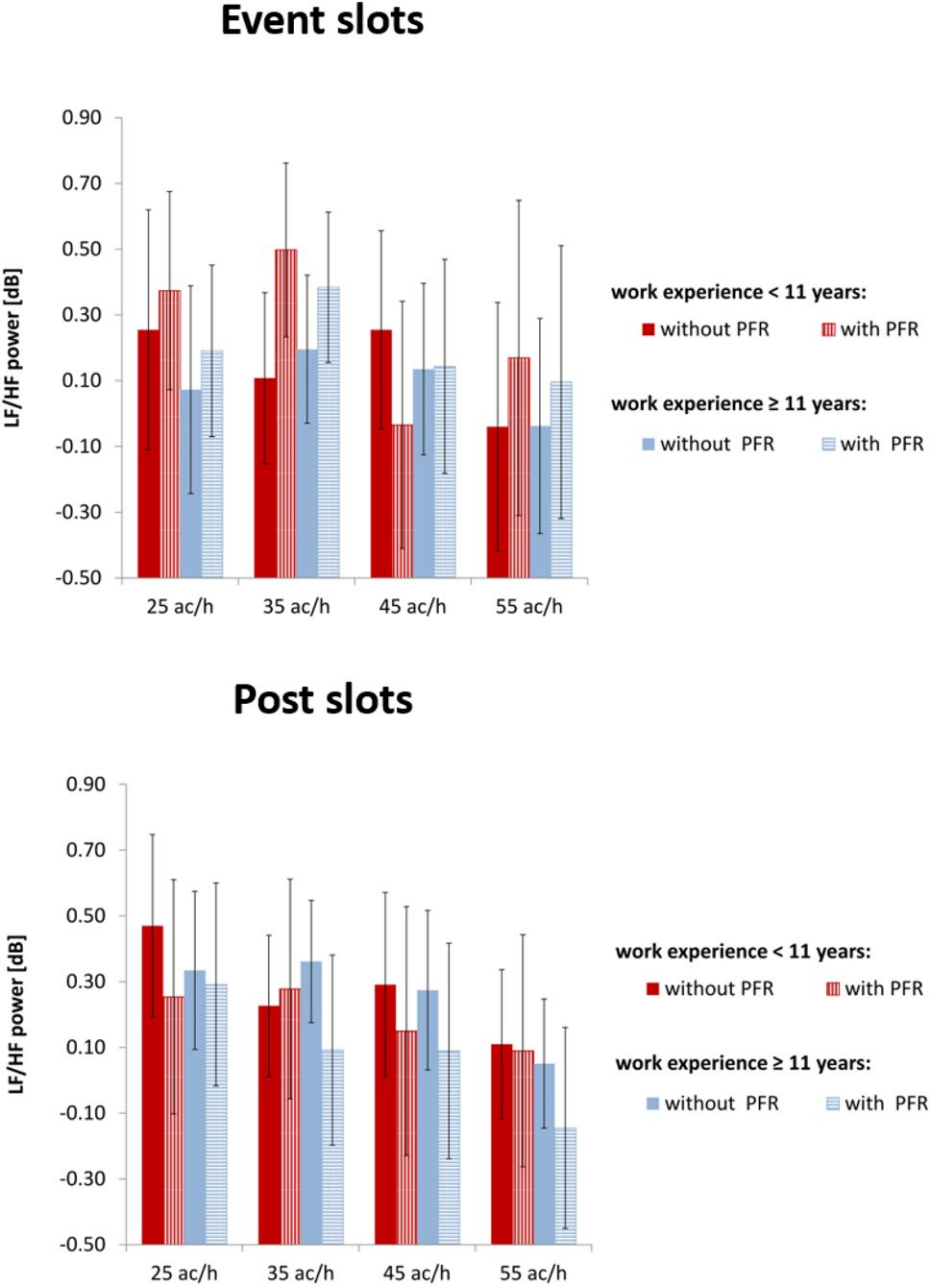
Comparison of work-experience groups. Means of the lg(LF/HF) for both time slots (top row: event slot, bottom row: post slot) during scenarios with and without priority-flight request (PFR) at different traffic loads (ac/h: aircraft per hour) for highly (blue) vs. less (red) experienced participants. Error bars indicate 95% confidence interval (Bonferroni corrected post hoc tests: ***p ≤ .001; **.001 < p ≤ .01; *.01 < p ≤ .05; error bars indicate 95% confidence interval)

Regarding the between-subject factor of work experience, no significant main effect could be found using the lg(LF/HF), neither during the event slots (F(1, 19) = 0.146, p = .706, η^2^ = 0.007) nor for the post slots (F(1, 19) = 0.237, p = .631, η^2^ = 0.012). Figure 3 shows the results. We refrained for further analysis regarding differences between work-experience groups as the between-subject factor was not significant.

**Fig. 4:**
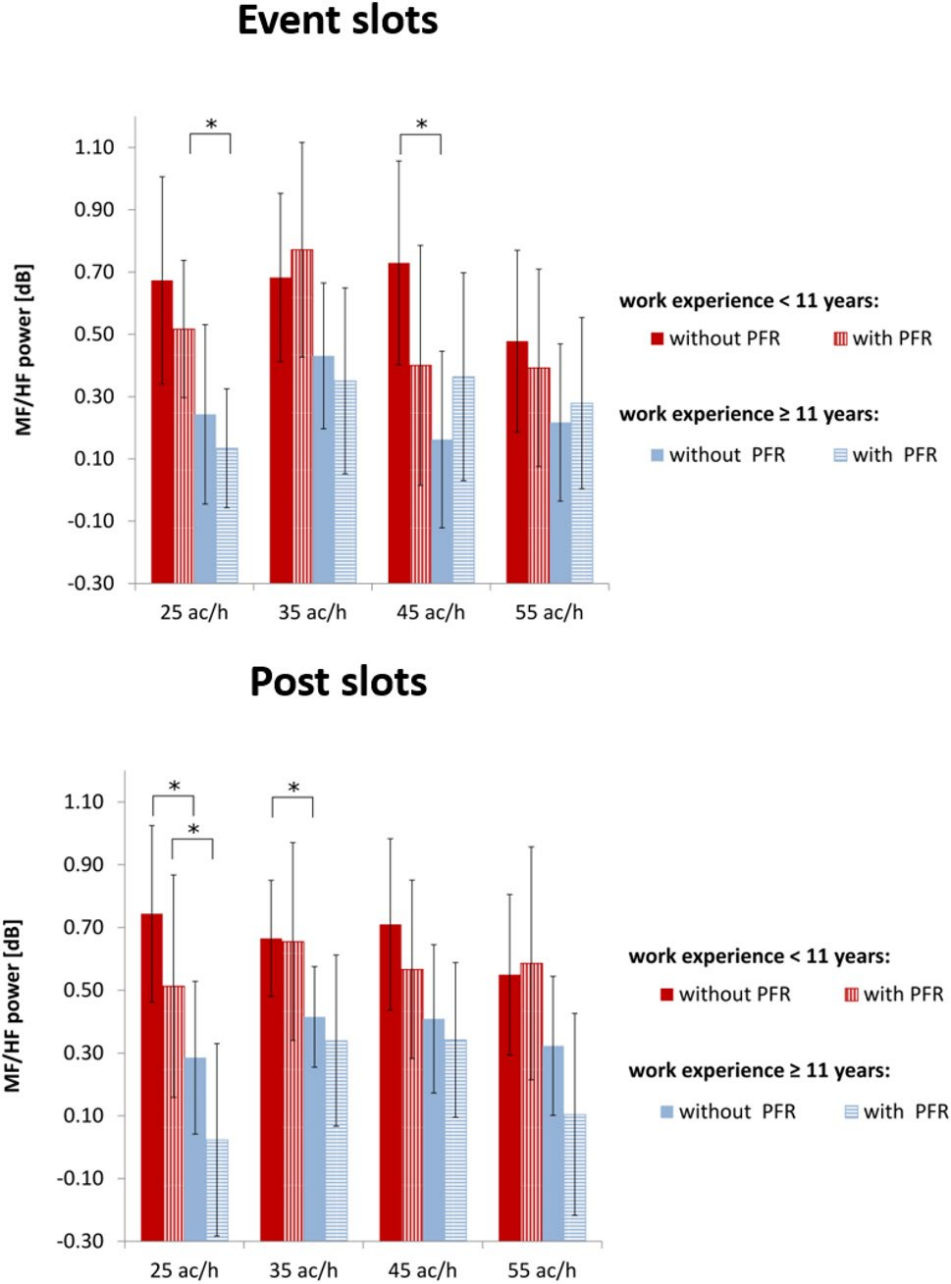
Comparison of work-experience groups. Means of the lg(MF/HF) for both time slots (top row: event slot, bottom row: post slot) during scenarios with and without priority-flight request (PFR) at different traffic loads (ac/h: aircraft per hour) for highly (blue) vs. less (red) experienced participants. Error bars indicate 95% confidence interval (Bonferroni corrected post hoc tests: ***p ≤ .001; **.001 < p ≤ .01; *.01 < p ≤ .05; error bars indicate 95% confidence interval)

The remaining cardiovascular biomarkers were more indicative. For both time slots, lg(MF/HF) showed a significant main effect for the between-subject factor of work experience, meaning that work-experience groups differed significantly (event slot: F(1, 19) = 6.44, p = .02, η^2^ = 0.253; post slot: F(1, 19) = 7.412, p = .013, η^2^ = 0.280). The results are shown in Fig. 4.

One-factorial ANOVAs for the work-experience factor were conducted, in order to find out at which scenarios the groups differed. During the event slots, lg(MF/HF) between the groups differed significantly for the 25 ac/h scenario with priority-flight request as well as for the 45 ac/h scenario without priority-flight request. During the post slots, lg(MF/HF) between the groups differed significantly for the 25 ac/h scenario with and without priority-flight request as well as for the 35 ac/h scenario without priority-flight request. For the more demanding scenarios no significant differences between groups could be obtained. The results are summarized in Table 3.

**Table 3:** Results of the ANOVAs for the lg(MF/HF) differences between work-experience groups across simulation conditions (ac/h: aircraft per hour) for event and post slots

Traffic load	Priority request	F	p	η^2^
Event slots				
25 ac/h	No	4.192	.055	.181
	Yes	7.578	**.013**	.285
35 ac/h	No	2.173	.157	.103
	Yes	3.738	.068	.164
45 ac/h	No	7.517	**.013**	.283
	Yes	0.022	.883	.001
55 ac/h	No	2.010	.172	.096
	Yes	0.318	.579	.016
Post slots				
25 ac/h	No	6.661	**.018**	.260
	Yes	4.781	**.041**	.201
35 ac/h	No	4.567	**.046**	.194
	Yes	2.515	.129	.117
45 ac/h	No	3.045	.097	.138
	Yes	1.565	.226	.076
55 ac/h	No	1.962	.177	.094
	Yes	4.213	.054	.181

Statistically significant values (*p* ≤ 0.05) are given in bold

Values of .001 are actually p ≤ .001

There was homogeneity of the error variances, as assessed by Levene’s test (p > .05)

For further evaluation of the within-subject factors (i.e., traffic load and priority-flight event) related to each work-experience group, we conducted two-factorial ANOVAs. No significant effects could be obtained for the lg(MF/HF) during the event slots neither for the highly-experienced nor for the less-experienced groups. During the post slots, we obtained a significant main effect for the priority-flight request for participants with high work experience (F(1, 11) = 9.816, p = .009, η^2^ = 0.471). The lg(MF/HF) was decreased during the priority-flight request scenarios.

**Fig. 5:**
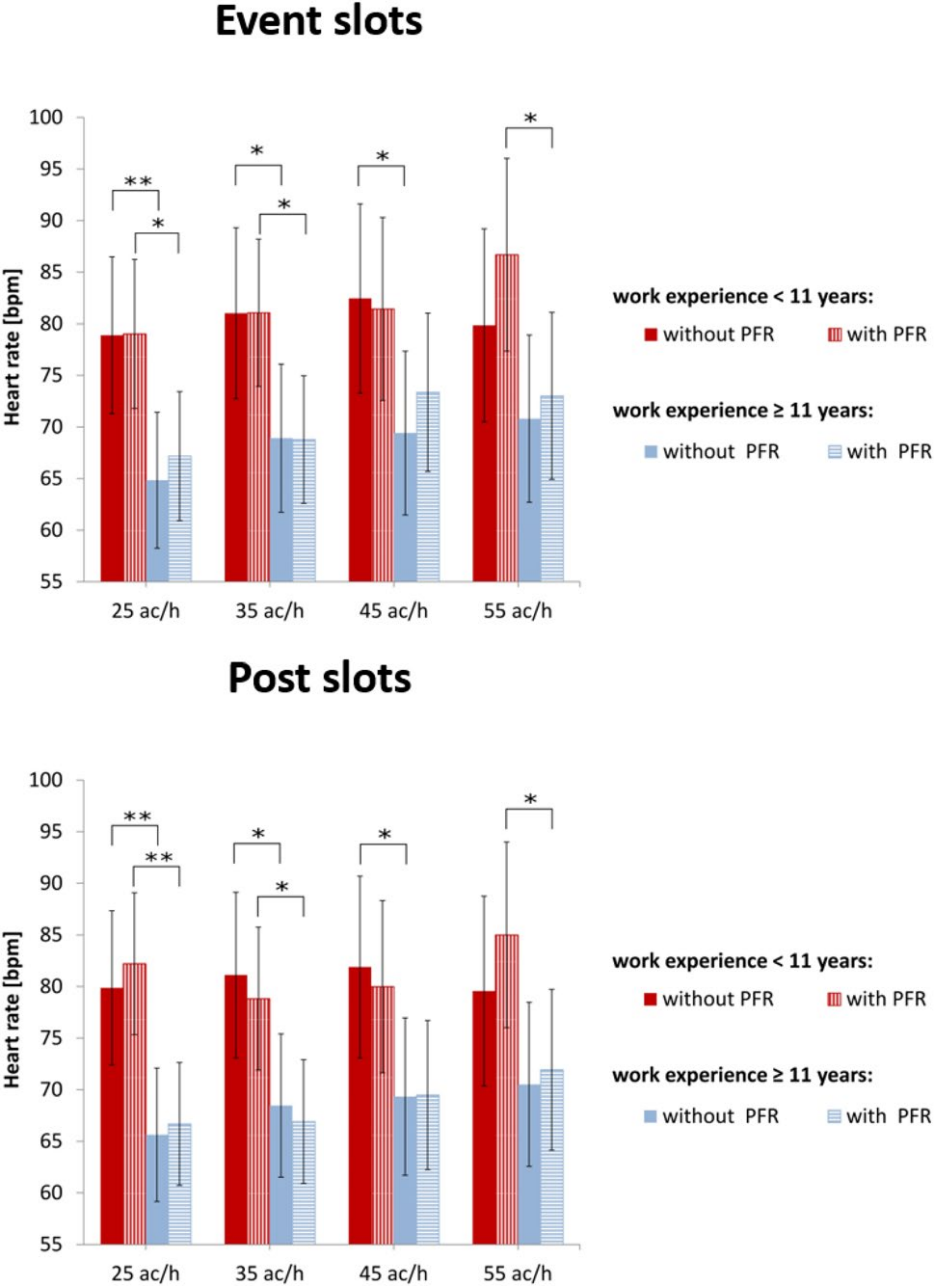
Comparison of work-experience groups. Mean values of heart rate for both time slots (top row: event slot, bottom row: post slot) during scenarios with and without priority-flight request (PFR) at different traffic loads (ac/h: aircraft per hour) for highly (blue) vs. less (red) experienced participants. Error bars indicate 95% confidence interval (Bonferroni corrected post hoc tests: ***p ≤ .001; **.001 < p ≤ .01; *.01 < p ≤ .05; error bars indicate 95% confidence interval)

Correspondingly, the heart rate revealed also a significant main effect of work experience (event slot: F(1, 19) = 5.921, p = .025, η^2^ = 0.237; post slot: F(1, 19) = 7.144, p = .015, η^2^ = 0.273). The results are shown in Fig. 5.

One-factorial ANOVAs for the work-experience factor were conducted, in order to find out at which scenarios the groups differed. During the event slots, heart-rate values between the groups differed significantly for almost all scenarios except for the 45 ac/h scenario with priority-flight request and the 55 ac/h scenario without priority-flight request. Similar results were obtained during the post slots and summarized in Table 4.

**Table 4:** Results of the ANOVAs for the heart-rate differences between work-experience groups across simulation conditions (ac/h: aircraft per hour) for event and post slots

Traffic load	Priority request	F	p	η^2^
Event slots				
25 ac/h	No	8.554	**.009**	.311
	Yes	6.745	**.018**	.262
35 ac/h	No	5.343	**.032**	.219
	Yes	7.414	**.014**	.281
45 ac/h	No	5.064	**.036**	.210
	Yes	2.076	.166	.098
55 ac/h	No	2.340	.143	.110
	Yes	5.379	**.032**	.221
Post slots				
25 ac/h	No	9.105	**.007**	.324
	Yes	12.799	**.002**	.402
35 ac/h	No	6.221	**.022**	.247
	Yes	7.399	**.014**	.280
45 ac/h	No	5.087	**.036**	.211
	Yes	3.962	.061	.173
55 ac/h	No	2.424	.136	.113
	Yes	5.281	**.033**	.218

Statistically significant values (*p* ≤ 0.05) are given in bold

Values of .001 are actually p ≤ .001

^a^Indicates Mauchly’s test of sphericity was significant (p < .05) and a Greenhouse–Geisser correction was made to degrees of  freedom

For further evaluation of the within-subject factors (i.e., traffic load and priority-flight event) related to each work-experience group, we conducted two-factorial ANOVAs. For participants with less work experience, no significant main effects for traffic load or priority-flight request could be obtained for the heart-rate values during the event or post slots. However, for event and post slots, we obtained a significant interaction effect of traffic load and priority-flight (event slot: F(3, 24) = 3.782, p = .023, η^2^ = 0.321; post slot: F(3, 24) = 3.542, p = .029, η^2^ = 0.306). The nature of this interaction is shown in Fig. 5. In particular during the high traffic-load scenario with 55 ac/h, heart rate increased during the scenario with a priority-flight request while it decreased during the scenario without the priority-flight request.

For participants with high work experience, we were able to obtain a significant main effect of traffic load for the heart-rate values of both time slots (event slot: F(3, 33) = 5.970, p = .002, η^2^ = 0.351; post slot: F(3, 33) = 3.289, p = .032, η^2^ = 0.230). Bonferroni-corrected post hoc tests revealed significant differences only during the event slots. Heart rate was significantly increased during high traffic with 55 ac/h compared to low traffic with 25 ac/h (p = .006). We were not able to obtain a significant main effect for the priority-flight request nor an interaction effect for none of the time slots.

## Discussion

In our study, we aimed in investigating cardiovascular biomarkers’ inherent timescales in mental workload assessment during simulated air traffic control tasks. We focused on air traffic controller’s working position for arrival management and conducted a study in a simulator with 21 participants. All of them completed in randomized order eight simulation scenarios with four different traffic-load levels, with and without a priority-flight request. If a priority-flight request was part of the scenario, it appeared around the 11th minute of simulation.

We considered the heart rate as well as HRV biomarkers from the frequency domain during event and post slots. The range of the frequency bands used was according to the definitions by Mulder (1992). In general, and as stated by Mulder and Mulder (1981) under mental load the total power decreased while heart rate increased. Analyses of heart rate, lg(LF/HF), and lg(MF/HF) were conducted for 3 min each in the event slot (around minute 11 to 14 for scenarios with and without priority-flight request) and post slot (17 to 20 min). During the event slots, we descriptively observed the tendency of both HRV biomarkers to decrease with increased traffic load while the heart rate increased. This was in accordance with other studies indicating that during high mental load HRV decreases while heart rate increases (Lei and Roetting 2011; Luft et al. 2009; Patel et al. 2011; Tjolleng et al. 2017). Regarding the priority-flight request results were ambiguous among our biomarkers. While the lg(MF/HF) revealed a slight suppression during scenarios with priority-flight request compared with the scenarios without priority-flight request, the lg(LF/HF) ratio showed the opposite behaviour. Heart rate behaved confirmatively to the lg(MF/HF) and was increased during scenarios with priority-flight request. Admittedly, none of the HRV biomarkers revealed significant differences neither between traffic load nor priority-flight request conditions. During the event slots, it was only the heart rate that showed significant differences between the low-traffic and the two higher-traffic conditions as well as between scenarios with and without priority-flight request. At this early stage, one would assume that heart rate is better suited to register mental workload than HRV biomarkers.

However, this assumption fluctuated when looking at the post slots. We observed a clear decrease of the HRV biomarkers with increasing traffic load as well as during scenarios with priority-flight request. Both HRV biomarkers were able to significantly discriminate between scenarios with and without priority-flight request. Heart rate, in general, increased with traffic load but was only able to significantly differentiate between two scenarios. During the post slots, heart rate was not any more able to significantly discriminate between priority-flight request conditions. To sum up, heart rate was better suited to assess workload differences during the event slots while the spectral HRV biomarkers were more indicative during the post slots. Additionally, computation of repeated-measures ANOVAs with three factors (i.e., priority-flight request, traffic volume, and time slot) indicated an interaction effect between the time-slot and priority-flight request factors that was highly significant for the lg(LF/HF) ratio (F(1, 20) = 22.184, p ≤ .001, η^2^ = 0.526) but failed to reach the significance level for the lg(MF/HF) ratio (F(1, 20) = 3.633, p = .071, η^2^ = 0.154). For the heart rate significant interaction effects were assessed between time-slot and priority-flight request factors (F(1, 20) = 5.107, p = .035, η^2^ = 0.203) as well as between time-slot and traffic-volume factors (F(3, 60) = 6.606, p = .001, η^2^ = 0.248). Nevertheless, we must note that our sample size might be too small for assessing insightful interaction effects between the three factors. Comparison of the radio-call activity of participants between the time slots did not show significant differences between event and post slots neither regarding the number of radio calls nor regarding radio-call duration for none of the eight scenarios. Hence, we assumed that differences between time slots were originated by cardiovascular biomarkers’ inherent timescales and not subject of variations in speaking.

Our third research question was concerned with the effect of participants’ prior work experience on the obtained cardiovascular biomarkers related to different load levels and time slots. No such effect could be found for the lg(LF/HF) for none of the time slots. Results from heart rate values and lg(MF/HF), however, indicated a significant between-subject effect of work experience. Heart-rate means of almost all scenarios differed significantly between less and highly experienced participants, in particular during event slots. Heart rate of less-experienced participants was increased and indicated a higher level of mental workload. This fact was in accordance with results of other studies (e.g., Paxion et al. 2014; Manzey et al. 2009) suggesting that mental workload decreases with a higher level of experience.

The lg(MF/HF) was able to better differentiate between the work-experience groups during the post slots. In particular, less-experienced participants had a significantly increased lg(MF/HF) during low-traffic scenarios when compared to the highly-experienced participants. This might indicate a stronger ability to relax during less demanding situations.

Examination of the within-subject factors traffic load and priority-flight request for each group separately added insight in our results. We start with the discussion of the results for the less-experienced participants. Especially during the event slots, heart rate revealed a significant interaction effect of traffic load and priority-flight request. During the high-traffic scenario, heart rate increased in the presence of a priority-flight request while it was decreased if the priority-flight request did not occur. We suggested that during the high-traffic scenario mental demands reached their maximum and less-experienced participants were mentally exhausted. In this situation, if a priority-flight request occurred, participants were actively requested to cope with the increased demands without giving up. We hypothesized that this was responsible for the abrupt increase of the heart rate and the significant interaction effect. For the less-experienced participants lg(MF/HF) did not yield any significant results for none of the time slots although it showed a decreasing tendency with increasing load and in scenarios with priority-flight request, in particular during the post slots. The lack of significant discrimination might be because most of our participants in the less-experienced group were employees of the DLR. They might have felt more familiar with the simulator environment, situation, and their colleagues conducting the experiment. Social expectations on them and accountability might be perceived or interpreted as lower compared to the professional air traffic controllers, leading to a more relaxed handling of the simulation scenarios and thus, flattering of the results.

Next, we discuss the effects of the within-subject factors for the highly-experienced participants as related to biomarkers’ inherent timescales. Heart rate of highly-experienced participants revealed a significant main effect of traffic load that was particularly prominent between the low and high traffic load during event slots. Heart-rate means were increased significantly with increased traffic load while the priority-flight request factor did not reach the significance level. However, the lg(MF/HF) ratio yielded a significant decrease for scenarios with a priority-flight request during the post slots. To sum up, heart rate of highly-experienced participants reacted significantly during event slots while the lg(MF/HF) was more indicative during the post slots. Considering each biomarkers’ inherent timescale (i.e., heart rate during the event slots and lg(MF/HF) during the post slots), heart rate of highly-experienced participants was sensitive to traffic-load variations while the lg(MF/HF) was sensitive to the priority-flight request. This might indicate that different cardiovascular biomarkers not only reveal different inherent timescales but also different diagnostics, thus different aspects of workload.

In this context, Matthews et al. (2015) suggested that there might be several workload indicators and not a universal one, similar to the fact that there was no general workload construct, either. However, the question arose if it was likely that only one of the measures is the “true” workload indicator, while the others related to different constructs. In addition, the authors emphasized that inter-individual differences should not be ruled out because of different sensitivity of individuals. Matthews et al. (2015) stated that as information-processing demands increased, multiple neuro-cognitive responses were possible (e.g., mobilization of resources, use of executive processes for monitoring and regulation of attention, control of compensatory workload, self-regulatory processes such as control of stress and emotion, and attempts of coping). If high mental task load triggered multiple responses, each of these responses could be considered as a workload indicator. This fact becomes more complex if biomarkers’ inherent timescales are to be considered. However, based on the results related to the differences between the less-experienced and highly-experienced participants as related to biomarkers’ inherent timescales, we were able to assess a significant effect of work experience on workload. Further research, certainly, should pay more attention in the selection of participants because work experience could be confounded by other aspects such as the habituation on the experimental environment.

## Conclusion

In our article we evaluated cardiovascular biomarkers regarding their ability to distinguish between conditions with different load levels and determined if different biomarkers have different inherent timescales in the assessment of workload. Additionally, we investigated the effect of participants’ prior work experience on the obtained cardiovascular biomarkers related to different load levels and time slots.

We focused on air traffic controller’s working position for arrival management, varied traffic load and the occurrence of an extraordinary event, and evaluated the heart rate as well as the lg(LF/HF) and lg(MF/HF). We were able to show that all three cardiovascular biomarkers were sensitive to workload differences, in particular when taking into account their immanent timescales. Heart rate reacted sooner and was most indicative during the time slot immediately after the relevant event while the biomarkers from the frequency domain reacted with a latency and were more pronounced during the post slots. During the particular time slots all biomarkers were able to significantly differentiate between conditions with and without priority-flight request. Moreover, heart rate and lg(LF/HF) significantly differentiated between low and high traffic conditions, in particular if biomarkers’ appropriate timescales were taken into account.

Work experience had a significant effect on the heart rate and lg(MF/HF) when considered in biomarkers’ relevant time slots. Interestingly, heart rate of highly-experienced participants was sensitive to traffic-load variations while the lg(MF/HF) was prone to workload variations arising from the priority-flight request. We suggested that these results indicate not only that different cardiovascular biomarkers reveal different latency in their response but also different diagnostics, thus different aspects of workload. The combination of cardiovascular biomarkers with different timescales can contribute to more stable and secure predictions about the individually experienced workload. Such a universally valid and multifaceted workload indicator would be particular suited for biofeedback and robust adaptive automation.

Study results can be seen as a preliminary investigation of cardiovascular biomarkers’ inherent timescales in mental workload assessment during simulated air traffic control tasks. For deriving an algorithm that integrates different timescales and their mappings to different workload facets, more research is needed. Future work should focus on different workload-relevant factors and examine their influence on the biomarkers and on their inherent timescales. Furthermore, a larger sample set would be beneficial for examining individual differences in biomarkers’ time behaviour.

To conclude, our study contributed to the investigation of inter-method dissociation by showing that workload responses among cardiovascular biomarkers were subject to temporal differences and might have accounted for these dissociations in prior research. Taking into account biomarkers’ inherent timescales as well as individual differences of participants could offer a way to gain more insight in the complexity of workload responses, thus providing a more stable and sound biofeedback.

## Data Availability

The conducted data used to support the findings of this study are restricted by the ethics committee of the Federal Institute for Occupational Safety and Health in order to protect participants’ privacy according to data-protection regulations. Data can be made available from the corresponding author upon request and after approval of the legal department for researchers who meet the criteria for access to confidential data.

## Appendix

See Tables 5, 6, 7, 8, 9 and 10.

**Table 5:** Mean and standard deviation (SD) values of used and additional cardiovascular parameters over 21 participants across simulation conditions (ac/h: aircraft per hour) with and without priority-flight request for event slots and post slots

	Traffic load	Priority request	Event slots		Post slots	
			Mean	SD	Mean	SD
lg(LF/HF)	25 ac/h	No	0.15	0.52	0.39	0.39
		Yes	0.27	0.43	0.28	0.50
	35 ac/h	No	0.16	0.37	0.30	0.31
		Yes	0.43	0.37	0.17	0.48
	45 ac/h	No	0.19	0.42	0.28	0.39
		Yes	0.07	0.53	0.12	0.53
	55 ac/h	No	− 0.04	0.53	0.08	0.32
		Yes	0.13	0.67	− 0.04	0.51
lg(MF/HF)	25 ac/h	No	0.43	0.51	0.48	0.46
		Yes	0.30	0.36	0.23	0.55
	35 ac/h	No	0.54	0.40	0.52	0.29
		Yes	0.53	0.53	0.47	0.47
	45 ac/h	No	0.41	0.54	0.54	0.41
		Yes	0.38	0.54	0.44	0.41
	55 ac/h	No	0.33	0.43	0.42	0.38
		Yes	0.33	0.45	0.31	0.57
Heart rate	25 ac/h	No	70.86	12.79	71.73	12.69
		Yes	72.25	11.74	73.34	12.41
	35 ac/h	No	74.10	13.10	73.88	12.91
		Yes	74.05	11.75	72.02	11.40
	45 ac/h	No	75.00	14.42	74.71	13.85
		Yes	76.82	13.05	73.98	12.82
	55 ac/h	No	74.68	13.85	74.39	13.63
		Yes	78.88	14.77	77.53	14.22
lg(LF)	25 ac/h	No	6.54	0.57	8.60	0.38
		Yes	6.83	0.43	6.90	0.50
	35 ac/h	No	6.54	0.31	8.43	0.26
		Yes	6.76	0.43	6.54	0.39
	45 ac/h	No	6.60	0.46	8.47	0.29
		Yes	6.56	0.46	6.51	0.42
	55 ac/h	No	6.62	0.34	8.48	0.45
		Yes	6.68	0.68	6.54	0.60
lg(HF)	25 ac/h	No	6.39	0.60	8.21	0.65
		Yes	6.56	0.45	6.62	0.68
	35 ac/h	No	6.38	0.46	8.13	0.39
		Yes	6.33	0.61	6.37	0.58
	45 ac/h	No	6.42	0.56	8.19	0.58
		Yes	6.50	0.59	6.39	0.52
	55 ac/h	No	6.66	0.65	8.41	0.66
		Yes	6.56	0.75	6.59	0.74
lg(MF)	25 ac/h	No	6.82	0.53	8.69	0.47
		Yes	6.86	0.40	6.86	0.54
	35 ac/h	No	6.92	0.40	8.65	0.33
		Yes	6.86	0.47	6.85	0.43
	45 ac/h	No	6.82	0.53	8.73	0.42
		Yes	6.88	0.40	6.83	0.43
	55 ac/h	No	6.98	0.50	8.83	0.44
		Yes	6.88	0.49	6.90	0.47
lg(SDNN)	25 ac/h	No	1.81	0.21	1.84	0.22
		Yes	1.84	0.18	1.86	0.21
	35 ac/h	No	1.77	0.16	1.74	0.15
		Yes	1.81	0.20	1.77	0.19
	45 ac/h	No	1.78	0.21	1.76	0.21
		Yes	1.82	0.18	1.78	0.15
	55 ac/h	No	1.83	0.23	1.85	0.21
		Yes	1.83	0.24	1.81	0.24

**Table 6:** Results of the ANOVAs for additional cardiovascular parameters across simulation conditions for the event and post slots

		F	p	η^2^
Event slots				
Traffic load	lg(LF)	0.506	.680	.025
	lg(HF)	2.712^a^	.089	.119
	lg(MF)	0.506	.679	.025
	lg(SDNN)	1.165^a^	.324	.055
Priority-flight request	lg(LF)	4.394	**.049**	.180
	lg(HF)	0.099	.756	.005
	lg(MF)	0.084	.775	.004
	lg(SDNN)	1.082	.311	.051
Traffic load and priority-flight request	lg(LF)	1.187	.322	.056
	lg(HF)	0.872	.461	.042
	lg(MF)	0.749	.527	.036
	lg(SDNN)	0.325	.807	.016
Post slots				
Traffic load	lg(LF)	5.247	**.003***	.208
	lg(HF)	2.719^a^	.074	.120
	lg(MF)	1.274	.291	.060
	lg(SDNN)	4.077^a^	**.023****	.169
Priority-flight request	lg(LF)	995.199	**.001**	.98
	lg(HF)	691.446	**.001**	.972
	lg(MF)	1434.496	**.001**	.986
	lg(SDNN)	0.145	.707	.007
Traffic load and priority-flight request	lg(LF)	1.567	.207	.073
	lg(HF)	0.679^a^	.500	.033
	lg(MF)	0.555	.646	.027
	lg(SDNN)	0.548	.652	.027

Statistically significant values (*p* ≤ 0.05) are given in bold

Values of .001 are actually p ≤ .001

*Bonferroni corrected post hoc tests indicated significant differences between 25 ac/h and 35 ac/h (p = .013), between 25 ac/h and 45 ac/h (p = .004), and between 25 ac/h and 55 ac/h (p = .038)

**Bonferroni corrected post hoc tests indicated significant differences between 25 ac/h and 35 ac/h (p = .010) and between 25 ac/h and 45 ac/h (p = .024)

^a^Indicates Mauchly’s test of sphericity was significant (p < .05) and a Greenhouse–Geisser correction was made to degrees of freedom

**Table 7:** Comparison of work-experience groups

	Traffic load	Priority request	Event slots				Post slots			
			Job experience (years)				Job experience (years)			
			< 11		≥ 11		< 11		≥ 11	
			Mean	SD	Mean	SD	Mean	SD	Mean	SD
lg (LF/HF)	25 ac/h	No	0.26	0.62	0.07	0.43	0.47	0.45	0.33	0.35
		Yes	0.37	0.34	0.19	0.49	0.25	0.29	0.29	0.62
	35 ac/h	No	0.11	0.26	0.20	0.44	0.23	0.29	0.36	0.32
		Yes	0.50	0.35	0.38	0.40	0.28	0.50	0.09	0.47
	45 ac/h	No	0.26	0.50	0.14	0.37	0.29	0.33	0.27	0.45
		Yes	− 0.03	0.56	0.14	0.53	0.15	0.40	0.09	0.63
	55 ac/h	No	− 0.04	0.48	− 0.04	0.58	0.11	0.26	0.05	0.37
		Yes	0.17	0.61	0.10	0.74	0.09	0.48	− 0.15	0.52
lg(MF/HF)	25 ac/h	No	0.67	0.57	0.24	0.40	0.74	0.43	0.29	0.38
		Yes	0.52	0.27	0.13	0.34	0.51	0.42	0.02	0.57
	35 ac/h	No	0.68	0.30	0.43	0.44	0.67	0.24	0.42	0.28
		Yes	0.77	0.48	0.35	0.50	0.66	0.38	0.34	0.50
	45 ac/h	No	0.73	0.52	0.16	0.43	0.71	0.33	0.41	0.43
		Yes	0.40	0.60	0.36	0.51	0.57	0.41	0.34	0.40
	55 ac/h	No	0.48	0.42	0.22	0.41	0.55	0.30	0.32	0.41
		Yes	0.39	0.34	0.28	0.52	0.59	0.37	0.10	0.63
Heart rate	25 ac/h	No	78.89	10.48	64.83	11.19	79.87	10.55	65.63	10.82
		Yes	79.03	10.64	67.17	10.14	82.21	8.92	66.68	10.46
	35 ac/h	No	81.02	9.91	68.92	13.12	81.11	10.32	68.46	12.28
		Yes	81.06	8.85	68.79	11.12	78.82	9.40	66.91	10.29
	45 ac/h	No	82.46	13.95	69.41	12.53	81.89	13.11	69.33	12.25
		Yes	81.44	13.96	73.36	11.72	79.99	13.37	69.48	10.84
	55 ac/h	No	79.85	12.75	70.81	13.86	79.56	13.71	70.51	12.76
		Yes	86.70	13.61	73.01	13.21	85.01	12.49	71.93	13.20
lg(LF)	25 ac/h	No	6.65	0.61	6.47	0.56	8.65	0.37	8.56	0.40
		Yes	6.93	0.43	6.76	0.43	6.89	0.55	6.90	0.49
	35 ac/h	No	6.56	0.32	6.52	0.31	8.40	0.17	8.45	0.31
		Yes	6.83	0.56	6.71	0.32	6.74	0.44	6.40	0.30
	45 ac/h	No	6.69	0.55	6.53	0.38	8.51	0.28	8.44	0.30
		Yes	6.57	0.39	6.56	0.51	6.69	0.32	6.37	0.44
	55 ac/h	No	6.68	0.30	6.57	0.37	8.53	0.39	8.44	0.50
		Yes	6.71	0.76	6.66	0.65	6.63	0.66	6.48	0.57
lg(HF)	25 ac/h	No	6.40	0.64	6.39	0.59	8.18	0.66	8.23	0.68
		Yes	6.55	0.29	6.57	0.55	6.64	0.61	6.61	0.75
	35 ac/h	No	6.45	0.42	6.33	0.50	8.18	0.33	8.09	0.45
		Yes	6.33	0.65	6.32	0.60	6.46	0.57	6.31	0.60
	45 ac/h	No	6.44	0.47	6.40	0.64	8.22	0.49	8.17	0.66
		Yes	6.60	0.55	6.41	0.64	6.54	0.48	6.28	0.54
	55 ac/h	No	6.72	0.51	6.61	0.76	8.42	0.48	8.39	0.79
		Yes	6.54	0.57	6.57	0.88	6.54	0.55	6.63	0.88
lg(MF)	25 ac/h	No	7.07	0.38	6.64	0.56	8.93	0.27	8.51	0.52
		Yes	7.07	0.29	6.71	0.40	7.15	0.45	6.63	0.49
	35 ac/h	No	7.14	0.25	6.76	0.42	8.84	0.17	8.51	0.36
		Yes	7.10	0.36	6.67	0.47	7.11	0.36	6.65	0.37
	45 ac/h	No	7.17	0.38	6.56	0.48	8.93	0.27	8.58	0.45
		Yes	7.00	0.42	6.78	0.36	7.10	0.30	6.63	0.41
	55 ac/h	No	7.20	0.23	6.83	0.59	8.97	0.24	8.72	0.53
		Yes	6.93	0.47	6.85	0.52	7.12	0.43	6.73	0.45
lg(SDNN)	25 ac/h	No	1.86	0.18	1.77	0.23	1.89	0.18	1.81	0.25
		Yes	1.91	0.14	1.79	0.20	1.92	0.18	1.82	0.22
	35 ac/h	No	1.81	0.13	1.74	0.17	1.79	0.13	1.70	0.15
		Yes	1.87	0.21	1.76	0.19	1.85	0.17	1.71	0.19
	45 ac/h	No	1.86	0.14	1.72	0.24	1.82	0.16	1.72	0.23
		Yes	1.88	0.14	1.78	0.20	1.87	0.12	1.71	0.14
	55 ac/h	No	1.87	0.14	1.81	0.28	1.88	0.13	1.82	0.26
		Yes	1.83	0.21	1.83	0.27	1.84	0.20	1.79	0.28

Mean and standard deviation (SD) values of used and additional cardiovascular parameters for event and post slots during scenarios with and without priority-flight request at different traffic loads (ac/h: aircraft per hour) for highly vs. less-experienced participants

**Table 8:** Results of the mixed-factorial ANOVAs for additional cardiovascular parameter regarding the between-subject factor of work experience for event and post slots

	F	p	η^2^
Event slots			
lg(LF)	0.819	.377	.041
lg(HF)	0.076	.785	.004
lg(MF)	8.100	**.010**	.299
lg(SDNN)	1.776	.198	.085
Post slots			
lg(LF)	1.010	.328	.050
lg(HF)	0.081	.779	.004
lg(MF)	8.738	**.008**	.315
lg(SDNN)	2.463	.133	.115

Statistically significant values (*p* ≤ 0.05) are given in bold

**Table 9:** Results of the ANOVAs for the lg(MF) differences between work-experience groups across simulation conditions (ac/h: aircraft per hour) for event and post slots

Traffic load	Priority request	F	p	η^2^
Event slots				
25 ac/h	No	3.981	.061	.173
	Yes	5.323	**.032**	.219
35 ac/h	No	5.676	**.028**	.230
	Yes	5.171	**.035**	.214
45 ac/h	No	9.629	**.006**	.336
	Yes	1.729	.204	.083
55 ac/h	No	3.175	.091	.143
	Yes	0.154	.699	.008
Post slots				
25 ac/h	No	4.694	**.043**	.198
	Yes	6.072	**.023**	.242
35 ac/h	No	6.779	**.017**	.263
	Yes	8.134	**.010**	.300
45 ac/h	No	4.400	**.050**	.188
	Yes	8.755	**.008**	.315
55 ac/h	No	1.759	.200	.085
	Yes	4.030	.059	.175

Statistically significant values (*p* ≤ 0.05) are given in bold

We refrained for further analysis regarding differences between work-experience groups for the remaining additional cardiovascular parameters as the between-subject factor was not significant (see Table 8)

**Table 10:** Results of further evaluation of the within-subject factors (i.e., traffic load and priority-flight event) related to each work-experience group for lg(MF) during the event and post slots

Work experience (years)		F	p	η^2^
Event slots				
> 11	Traffic load	1.092	.366	.090
	Priority-flight request	0.427	.527	.037
	Traffic load and priority-flight request	1.149	.344	.095
	Post slots			
	Traffic load	1.711^a^	.206	.135
	Priority-flight request	852.320	**.001**	.987
	Traffic load and priority-flight request	0.487	.694	.042
Event slots				
< 11	Traffic load	0.063	.979	.008
	Priority-flight request	3.239	.11	.288
	Traffic load and priority-flight request	0.856	.477	.097
	Post slots			
	Traffic load	0.173	.914	.021
	Priority-flight request	623.043	**.001**	.987
	Traffic load and priority-flight request	0.142	.934	.017

Statistically significant values (*p* ≤ 0.05) are given in bold

We refrained for further analysis regarding factor differences within work-experience groups for the remaining additional cardiovascular parameters as the between-subject factor was not significant (see Table 8)

Values of .001 are actually p ≤ .001

^a^Indicates Mauchly’s test of sphericity was significant (p < .05) and a Greenhouse–Geisser correction was made to degrees of freedom
